# Identifying phonological processing deficits in Northern Sotho-speaking children: The use of non-word repetition as a language assessment tool in the South African context

**DOI:** 10.4102/sajcd.v63i2.145

**Published:** 2016-05-20

**Authors:** Carien Wilsenach

**Affiliations:** 1 Department of Linguistics, University of South Africa, South Africa

## Abstract

Diagnostic testing of speech/language skills in the African languages spoken in South Africa is a challenging task, as standardised language tests in the official languages of South Africa barely exist. Commercially available language tests are in English, and have been standardised in other parts of the world. Such tests are often translated into African languages, a practice that speech language therapists deem linguistically and culturally inappropriate. In response to the need for developing clinical language assessment instruments that could be used in South Africa, this article reports on data collected with a Northern Sotho non-word repetition task (NRT). Non-word repetition measures various aspects of phonological processing, including phonological working memory (PWM), and is used widely by speech language therapists, linguists, and educational psychologists in the Western world. The design of a novel Northern Sotho NRT is described, and it is argued that the task could be used successfully in the South African context to discriminate between children with weak and strong Northern Sotho phonological processing ability, regardless of the language of learning and teaching. The NRT was piloted with 120 third graders, and showed moderate to strong correlations with other measures of PWM, such as digit span and English non-word repetition. Furthermore, the task was positively associated with both word and fluent reading in Northern Sotho, and it reliably predicted reading outcomes in the tested population. Suggestions are made for improving the current version of the Northern Sotho NRT, whereafter it should be suitable to test learners from various age groups.

## Introduction

### Language assessment in South African languages

The provision of effective services to patients from diverse linguistic backgrounds is a big challenge within the South African healthcare system (Mosdell, Balchin & Ameen, 2010; Pascoe & Norman, 2011; Van Dulm & Southwood, 2014). Almost 20 years after becoming an officially multilingual country, the provision of speech-language therapist (SLT) services to a linguistically diverse population remains problematic. The majority of SLT professionals speak English or Afrikaans (or both) and have little or no knowledge of the official African languages (Bornman, Sevcik, Romski & Pae, 2010; Gerber, 2009; Van Dulm & Southwood, 2014). This is a particularly grave concern, given the fact that in SLT services ‘language’ is not only used as a means of communication, but is the reason why a person is seeking help.

Formal assessment of a patient’s linguistic profile and linguistic problems provide a baseline for intervention, but is compromised if a patient is assessed in a language that is not his or her first language. This is especially true for children. In South Africa, the majority of child language assessment measures used are in English and have been standardised in the United Kingdom or the United States of America. As a result, (*ad hoc*) translators or translations are often used by South African SLTs in clinical encounters. In a national survey conducted by Van Dulm and Southwood (2014), which investigated child language assessment and intervention practices in South Africa, less than half of SLTs who participated felt that commercially available or translated language assessment instruments are linguistically appropriate for the South African context. Only about one-third of the SLTs thought that the instruments they use are culturally appropriate. Thus, SLTs are aware that their lack of knowledge of the native languages of their patients, combined with the lack of standardised language tests in African languages, in fact jeopardise language assessment and intervention results. Barrett, Khoza-Shangase and Msimang (2012) note that ‘this awareness should range from case history taking to the actual assessment to the test material used in the assessment, and therefore more emphasis should to be placed on development of assessment and intervention materials in all languages’.

In response to the need for assessment materials in South African languages, the current study explored the usefulness of developing a non-word repetition task (NRT) in Northern Sotho (one of the official languages of South Africa). Internationally, the NRT is used widely to access both normal and impaired language acquisition. The NRT is particularly associated with diagnosing children with speech-language disorders such as specific language impairment (SLI) and dyslexia, but has become a widely used tool in the field of speech-language pathology (also in the population at large); the reason being that non-word repetition closely matches the phonological component of word learning and is associated with phonological working memory (PWM), a construct which is crucially important in the attainment of language and literacy skills (Dollaghan & Campbell, 1998; Gathercole, Willis, Baddeley & Emslie, 1994). In this study, data gathered with a Northern Sotho NRT (NS NRT) will be presented. Despite the routine use of the NRT as a clinical assessment instrument in other parts of the world, such tasks have not yet been standardised for any of the African languages spoken in South Africa. The aim of this study was to determine the effectiveness of a NRT in determining different levels of phonological processing in Northern Sotho-speaking children, and to investigate whether performance on a NS NRT reliably predicts reading performance in Northern Sotho children. Given the high occurrence of reading failure in South African schools, developing tests which measure linguistic skills associated with reading is critically important.

## Literature review

### Phonological processing

The broad construct *phonological processing* consists of ‘three separate but interrelated phonological abilities, namely phonological awareness, phonetic coding in working memory, and phonological recoding in lexical access’ (Claessen, Leitão, Kane & Williams, 2013, p. 472). In its broadest sense, phonological processing has been defined as the ability to encode, store, retrieve, and reproduce sounds (De Bree, 2007). More specifically, phonological processing involves the encoding of auditory (linguistic) signals, the temporary storage of these signals, the retrieval of stored signals, and the planning involved in reproducing these signals (Chiat, 2001; Snowling, Nation, Moxham, Gallagher & Frith, 1997).

The focus of this article is on PWM and therefore ‘phonological awareness’ and ‘phonological recoding’ will not be discussed further. PWM is a basic cognitive skill that underlies all successful auditory language processing. Specifically, it underlies the online cognitive processing that allows the segmentation of the incoming speech stream and the subsequent development of accurate phonological representations of sounds, and (later on) words. In infants and young children, the PWM has to store strings of sounds representing words that are unfamiliar sound sequences when the child hears them for the first time. The quality of a child’s PWM is thought to affect the accuracy and efficiency with which the phonological representations of words are converted from short-term to long-term memory. Thus, PWM is very closely related to vocabulary acquisition, whereas other measures of working memory may be more related to language comprehension and syntactic processing (Gathercole, 2006).

The best-known model of working memory has been proposed by Baddeley (1999, 2003). In this model, PWM is depicted as a subcomponent, i.e. the ‘phonological loop’, of working memory. The other subcomponents of working memory include the visuospatial sketchpad, the central executive, and the episodic buffer. The phonological loop consists of a phonological store (that retains all verbal information that has to be processed or produced for a short period) and an articulatory loop (that rehearses and invigorates the verbal information in the phonological store to ensure that it does not fade away before being processed accurately) (Baddeley, 1999). The phonological loop is active in the acquisition of new vocabulary, in general problem-solving, numerical problem solving, and in remembering instructions. The central executive incorporates information from the phonological loop, the visuospatial sketchpad, and the episodic buffer; and guides the conversion of information from the phonological loop to long-term memory. The central executive is further implicated in multitasking, selective attention, suppression of irrelevant information, and temporary activation of long-term memory. PWM is generally assessed with tasks that tap into the phonological loop and the central executive, such as the digit span forward and backward task and the NRT.

PWM is believed to also affect the acquisition and processing of written language (Catts & Kamhi, 1999; Geva & Siegel, 2000; Nithart *et al*., 2011), because the ability to decode a word (or syllable) into phonemes (which is necessary for learning to read) requires the PWM system to store the entire phonological representation of a word while the constituent phonemes of that word are first disjointed and then sequenced back into the correct order by the learner. Poor PWM may lead to unstable phoneme representations in a person’s long term memory, which in turn may affect the acquisition of the phoneme-grapheme correspondences of a language negatively.

Numerous studies suggest that deficits in PWM play a causal role in the phonological processing limitations that manifest in diverse clinical populations. PWM deficits have been documented in developmental genetic language disorders, such as SLI (Claessen, *et al*., 2013; Conti-Ramsden & Durkin, 2007; Gathercole, 2006), dyslexia (Laasonen, Lehtinen, Leppamaki & Tani, 2010; Ramus, 2003; Spironelli, Penolazzi, Vio & Angrilli, 2006) and in other genetic syndromes such as Down’s syndrome (Jarrold & Baddeley, 2001; Numminen, Service, Ahonen & Ruoppila, 2001). The cause(s) for a PWM deficit itself is the topic of much neurobiological research. Genetic-molecular studies using non-word repetition ‘have reported linkage to regions of interest on chromosomes 16 and 19 for children with language impairment’ (Shriberg, *et al*., 2009). Other genetic regions of interest are chromosome 3 and chromosome 6. So far, one gene, namely *ROBO1* (situated on chromosome 3), has been linked specifically to the functioning of the phonological loop of the PWM system (Bates, *et al*., 2011).

### Non-word repetition tasks

Two decades ago Gathercole *et al*. (1994) observed that children who experience learning difficulties and who fail to progress in a formal schooling environment, often have difficulties in remembering spoken language even for short periods of time. This limitation manifests itself as the apparent inability of a child to attend to and carry out simple instructions. In an attempt to discover the precise role of phonological memory in children’s language development, Gathercole *et al*. (1994) developed the *Children’s test of non-word repetition* (*CNRep*). In their initial study, the CNRep was administered to more than 600 children, and the test developers found that the correlations between non-word repetition and language skills were consistently stronger than those between auditory digit span (which also contains a significant PWM component) and language skills.

In a NRT, a person has to repeat ‘pseudo’ words. Pseudo words are word forms that *could have been* lexical items in a particular language, in that they follow the morphophonological rules of that language, but which do not exist. Non-word repetition is more closely associated with linguistic abilities such as vocabulary knowledge, understanding of spoken language, reading ability, and later school performance than the digit span task (Gathercole, 2006; Gathercole & Baddeley, 1996; Gathercole *et al*., 1994). Because a NRT consists of novel items, which no child would have encountered prior to testing, it does not disadvantage children who received impoverished linguistic input (and thus have smaller vocabularies).

Even though NRTs are often said to measure PWM, such tasks measure a different aspect of phonological memory than digit or word span tasks. NRT is believed to entail the phonological analysis and temporary storage of *novel* phonological representations, with an individual being asked to recall a representation that at best mimics existing words (Nithart *et al*., 2011). Digit and word span, on the other hand, entail the processing of representations that already have a stored form in long-term memory – this stored information assists an individual in the analysis and temporary storage of phonological information. Thus, non-word repetition is thought to represent a clearer view of an individual’s phonological processing capacity in real time, and is more likely to detect inefficiencies in the phonological loop of PWM than digit or word span tasks.

NRTs are typically designed to contain both simple and complex words. The complexity of non-words is determined by two factors:

- the phonotactic structure of the word (e.g. a word containing consonant clusters is more complex than a word containing no consonant clusters)
- the length (e.g. a four syllable word is more complex than a two syllable word).

Clinical populations, such as dyslexics, children with SLI, people living with Down’s syndrome, and cochlear implant patients have all been found to be impaired on NRTs. These groups score significantly lower on non-word repetition than their age/language-matched normally developing peers. On the basis of this, and seeing that reading-disabled people also experience phonological awareness and phonological recoding impairments, the *phonological deficit hypothesis* (Ramus, 2001; Snowling, 2001; Vellutino, Fletcher, Snowling, & Scanlon, 2004) became one of the most influential theories in explaining reading disability in clinical populations. Generally speaking, non-word repetition deficits appear to remain present in reading-disabled individuals, even into adolescence and adulthood (De Bree, 2007). This is less true for digit span and word span tasks, which confirms that, although these tasks are related, they tap into different aspects of phonological processing.

There is no agreement as to whether the NRT can be used to predict reading ability in the population at large. Bishop, Adams and Norbury (2004, p. 96) are adamant that non-word repetition *does not* predict reading outcome in the general population at large. According to these scholars:

A prediction…is that a sample of children with reading disability recruited by general population screening would not be expected to contain many cases with poor non-word repetition. However, …we would expect to see poor NRT scores in dyslexic samples who were selected for genetic linkage studies…

Bishop *et al*. (2004) are of the opinion that reading difficulties in non-clinical groups are caused by environmental factors such as poverty, inadequate education, and social factors such as the age and education of a child’s mother and the child’s position in the family. Although all of these environmental factors probably contribute to the low literacy levels of South African learners, the causal role of underdeveloped phonological processing skills and vocabulary skills (see Wilsenach, 2015), which are strongly associated with non-word repetition, remains unclear in the South African context.

Studies on the use of the NRT in African languages are very limited. Veii and Everatt (2005) investigated predictors of word reading among Grade 2–5 Herero-English bilingual children. They included Herero and English phoneme recognition, PWM, a Herero and English NRT, Herero and English rapid naming, and Herero and English word reading as measures. Phonological skills in Herero and English (such as phoneme awareness and non-word repetition) reliably predicted reading ability in both languages. Alcock, Ngorosho, Deus and Jukes (2010) studied the relationship between phonological awareness and literacy in Swahili-speaking children. They investigated implicit and explicit phonological awareness skills and literacy in Tanzanian school-going and non-school-going children between the ages of 7 and 10. Alcock *et al*. (2010) found that children who could not read had poorer phonological awareness skills than those who could, except in counting syllables, counting sounds, and in NRT (however, there might have been a ceiling effect in the NRT).

Wilsenach (2013) assessed phonological processing (including syllable awareness, non-word repetition, and digit span) in two groups of Northern Sotho-speaking third graders. One group received their schooling in their home language (Northern Sotho) from Grade 1–3, whereas the other group received their schooling in English from the beginning of Grade 1. Contrary to Bishop *et al*.’s (2004) prediction, Northern Sotho non-word repetition reliably discriminated between the two groups of normally developing Northern Sotho-speaking children. However, Wilsenach (2013) acknowledged that the study was limited – primarily because of the small sample (50 overall), and recommended that a larger sample needs to be tested.

The present study set out to measure phonological processing, specifically non-word repetition, memory for digits and reading in 120 Northern-Sotho third graders. In order to evaluate the effect of the Language of Learning and Teaching (LoLT) on the learners’ ability to process sounds in their mother tongue, half of the learners received their schooling in Northern Sotho, and the other half received their schooling in English.

## Research questions and hypotheses

The following research questions were addressed in this study:

- Does the NS NRT used here discriminate between Northern Sotho-speaking children with good and poor PWM skills?
- Does the LoLT affect the performance of Northern Sotho-speaking children on a NS NRT and on an English NRT?
- Does the NS NRT used here correlate with other measures that tap into PWM, such as digit span and English NRT?
- Does the NS NRT used here predict word reading and fluent reading in Northern Sotho-speaking children?

On the basis of Wilsenach (2013), it is hypothesised that learners who received instruction in Northern Sotho will fare better in the NS NRT. Furthermore, it is hypothesised that, in the sample as a whole, the NS NRT will correlate with digit span, with English non-word repetition, and with Northern Sotho reading ability. Finally, it is predicted that learners who received instruction in English will display enhanced phonological processing abilities in English, resulting in higher scores on the English NRT.

## Ethical considerations

The learners who participated in this project were recruited on a voluntary basis. Ethical clearance was obtained from the relevant educational authorities, and learners participated only if their caregivers signed an informed consent form (which was provided in both Northern Sotho and English) and if they themselves agreed to participate before the start of the testing session. Learners’ identities were kept confidential, and data obtained from this project were not disclosed to any third party. There were no physical risks involved in taking part in the research. Psychological risks were minimised by discontinuing any assessment that proved too difficult for a particular learner.

## Research method and design

### Research setting

The current study was conducted in two primary schools located in a high-poverty suburb of Pretoria (one of the capital cities of South Africa). Northern Sotho is the most common home language in this suburb, but several other African languages occur in the research area. Some schools within the area use Northern Sotho as LoLT from Grade R until the end of Grade 3, after which the curriculum is delivered via English. In the present study, Northern Sotho was used as LoLT in one of the schools; the other school followed a straight for English language policy.

### Participants

One hundred and twenty Grade-3 learners^1^ were selected to participate in the study, using a purposive and convenience sampling technique. In order to participate in the study, learners had to speak Northern Sotho as their home language. The participants were made up of two groups: Group 1 (*N* = 60; mean age 8.8) was instructed in English (with Northern Sotho being taught as an additional language), whereas Group 2 (*N* = 60; mean age 8.8) was instructed in Northern Sotho (with English being taught as an additional language). There was no significant difference between the groups in terms of age (*t* = -0.04; *p* = 0.96). Generally speaking, learners from both groups came from poor households, as evidenced by the fact that both schools have feeding schemes for learners (the children in Group 1 attended a quantile 2 school, whereas the children in Group 2 attended a quantile 1 school).^2^ Group 1 contained 35 girls; Group 2 contained 29 girls.

### Research design and materials

The research was conducted as a cross-sectional quantitative study. The participants were tested individually during a single session. Various phonological skills (not all reported on here) and reading were tested. The research was conducted during a 5-week period in the second term of the school year. All the tasks were presented to the learners using a computer and headphones – this ensured that the auditory presentation happened accurately, and with consistent rate and consistent intonation. Performance was scored online, but a digital recording of each test session was made in order to be able to check a learner’s responses.

PWM were measured with a NS NRT, an English NRT, and an English digit span task.^3^ The NS NRT was similar to the task reported on in Wilsenach (2013). It was designed following most of the criteria set out by Dollaghan and Campbell (1998), including:

- neither the non-words nor their constituent syllables corresponded to lexical items
- the non-words included phonemes and syllable types that are acquired early in development
- The non-words were phonotactically possible in Northern Sotho.

The NS NRT consisted of 20 test items, ranging from four syllables (e.g. *sepokari*) to seven syllables (e.g. *nasibhekarabile*) in length. The test comprised five items at each syllable length. The non-words were pre-recorded by a native speaker of Northern Sotho and were presented in the same order to each of the participants. The 20 test items are included in Appendix A.

English NRT was measured with subtest 5 of the *Comprehensive Test of Phonological Processing* (CTOPP) (Wagner, Torgesen & Rashotte, 1999). This NRT proceeded from 1-syllable non-words to 10-syllable non-words. PWM was further assessed with subtest four (memory for digits) from the CTOPP. The participants had to recall strings of digits in English, as presented on the CTOPP CD. The memory for digits test started with strings of two digits, and progressed to strings with nine digits. The English NRT and the memory for digits test were discontinued after three consecutive incorrect responses. The rationale for including the English NRT and the memory for digits task here is that the scores obtained on the NS NRT should correlate with these measures, if the NS NRT indeed taps into phonological processing/PWM abilities.

Word reading in Northern Sotho was tested with a tailor made word reading task, consisting of 30 items (in order of increasing length and difficulty; see Appendix B). Northern Sotho text reading was assessed by measuring the amount of words that learners could read accurately in one minute, using a grade appropriate reader. Word reading in English was assessed with the *Diagnostic test of word reading processes* (FRLL, Institute of Education, 2012), which consists of three subsections (non-word reading, exception word reading, and regular word reading). Only regular word reading will be reported on here, as this subsection of the test best compared with the Northern Sotho word reading test. English text reading was assessed by measuring the amount of words that learners could read accurately in one minute, using a grade appropriate reader.

### Data analysis and statistical procedures

Raw scores were calculated for every participant on each of the measures. The raw scores on the NS NRT and on the Northern Sotho word reading test were transformed into percentages, and based on these scores a mean raw score and mean percentage were calculated for the sample as a whole, as well as for the two groups. The raw scores on the English NRT and on the memory for digits test were converted to standard scores (SSs), using the age norms in Wagner *et al*. (1999). The raw scores on the English word reading test were used in the statistical analyses, as the GL assessment manual only provides SS for the composite word reading raw score (consisting of all three subsections of the test). Raw scores for Northern Sotho text reading and English text reading were calculated by counting the number of words that a child read aloud in 1 min, and subtracting the number of incorrectly read words from this total.

The distribution of the test scores on the NS NRT was tested with the Shapiro–Wilk test, and was further analysed via a visual inspection of the data. Seeing that the NS NRT is not a standardised test, it was important to confirm that the test scores were distributed normally. The effect of LoLT on Northern Sotho phonological processing ability, as well as between-group differences (in terms of NS NRT, English NRT, and PWM, and reading) were tested using a multivariate analysis of variance (MANOVA) (GLM option multivariate). *Group* was entered as an independent variable in the model, whereas *NS NRT, English NRT, Digit span, NS Word reading, NS Text reading, English word reading*, and *English text reading* were entered as dependent variables. Pearson correlations were conducted in order to determine the relationship between NS non-word repetition and related phonological constructs, such as memory for digits and English non-word repetition. Hierarchical multiple regressions (method *enter*) were conducted to determine the contribution of NS non-word repetition to NS reading.

## Results

### Distribution of Northern Sotho non-word repetition task scores

The descriptive statistics for the NS NRT are given in Table 1.

**TABLE 1:** Descriptive statistics for Northern Sotho non-word repetition task.

Measure (*N* = 120)	Min score	Max score	Mean score	SD	Percentiles						

					5	10	25	50	75	90	95
-	1	19	11.46	3.85	5	6	9	12	14	16	17.95

Min, minimum; Max, maximum; SD, standard deviation.

**FIGURE 1:**
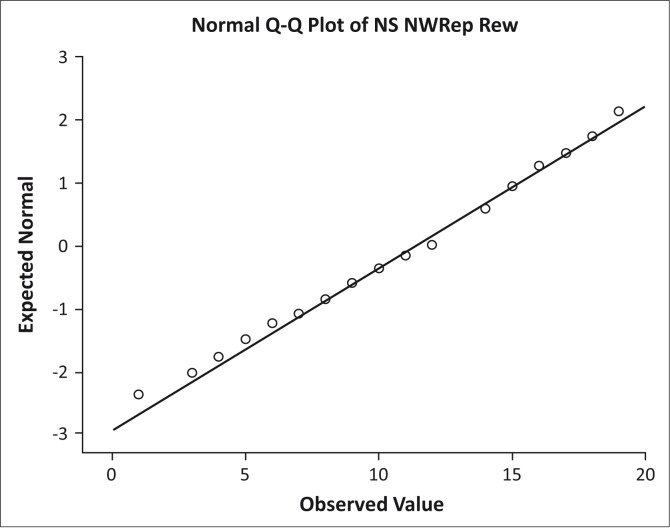
Normal Q-Q plot of NS NRT scores.

A Shapiro–Wilk test, performed on the collapsed data, yielded a non-significant result, indicating that the NS NRT test scores were distributed normally (*W*(120) = 0.98, *p* = 0.058). Both skewness (-0.31; SE = 0.22) and kurtosis (-0.36; SE = 0.49) were negative, suggesting a build-up of high scores, but the associated *z*-scores for both skewness and kurtosis (calculated by dividing these values by their standard errors) were smaller than 1.96, indicating no significant problems with either skew or kurtosis. Further support for the claim that the NS NRT test scores were distributed normally is derived from the normal quantile-quantile (Q-Q) plot (Figure 1), in which the plotted quantiles fall very closely to the diagonal line (which represents a perfect normal distribution).

**FIGURE 2:**
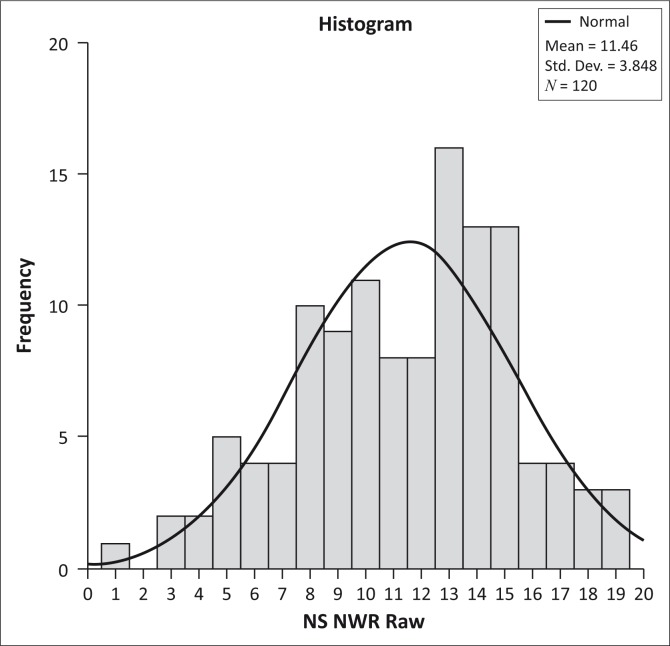
Distribution of NS NRT scores in the tested sample.

The histogram in Figure 2 confirms a (relatively) flat and light-tailed distribution of the NS NRT scores, but also indicates a slight build-up of higher scores; with a peak at raw score 13 (i.e. 65% correct responses to the test). The visual inspection of the data seems to suggest that a peak at 11 or 12 would fit the normal distribution curve better.

Nevertheless, for the purposes of this study, the non-significant result of the Shapiro–Wilk test was seen as sufficient evidence that the scores obtained in the NS NRT were normally distributed, and hence the rest of the statistical analyses were conducted using parametric tests.

### Main effects and between group differences

The GLM model (option Multivariate) established a significant main effect for *Group* (*F*[3,116] = 9.67, *p* = 0.000). Following this significant effect, tests of between-subjects effect were conducted, which showed that *Group* had a significant effect on English NRT (*F* = 14.13, *p* = 0.000), but no effect on Digit span or NS NRT. Pairwise comparisons (Tukey–Bonferroni *post hoc* tests) confirmed that there were no significant differences between the groups in terms of Digit span or NS NRT, but that Group 1 significantly outperformed Group 2 in the English NRT. Furthermore, Group 1 performed significantly better on both English reading measures, whereas Group 2 performed significantly better on both the Northern Sotho reading measures. The mean scores and test statistics for the measures *Digit span, NS NRT, English NRT, Northern Sotho word reading, Northern Sotho text reading, English word reading*, and *English text reading* are presented in Table 2.

**TABLE 2:** Mean raw score and SS for *Digit span*, Mean percentage for *Northern Sotho non-word repetition task* (NS NRT), Mean raw score and SS score for *English NRT*, Means for *word reading* and raw score for *fluent reading*.

Measures	Group 1 (*N* = 60)		Group 2 (*N* = 60)		Test statistics	
		
	Mean	(SE)	Mean	(SE)	*F*	*p*
**Phonological working memory** Digit span Raw score SS NS Non-word repetition Percentage correct English Non-word repetition Raw score SS	15.43 8.45 54.50 17.23 10.03	*0.37* *0.35* *2.47* *0.43* *0.43*	14.61 7.65 60.08 14.97 7.83	*0.37* *0.35* *2.47* *0.43* *0.43*	2.44 2.57 2.56 14.14 13.13	0.12 0.11 0.11 0.001* 0.001*
**Northern Sotho reading** Word reading % correct Text reading raw score	45.56 20.38	*4.66* *2.80*	67.11 29.05	*4.66* *2.80*	10.70 4.83	0.001* 0.03*
**English reading** Word reading raw score Text reading raw score	11.6 66	*0.99* *5.31*	8.27 36.4	*0.96* *5.17*	5.84 15.93	0.017* 0.000*

SS, standard score.

* Significant at the 0.05 level.

### Correlations and multiple regressions

Pearson correlations (two-tailed) indicated that *NS NRT* was significantly correlated with *Digit span, English NRT, NS word reading, NS text reading* and *English word reading*. English NRT was significantly correlated with both English reading measures, but showed no correlation with the NS reading measures. Digit span was significantly associated with all the reading measures in both languages. The *r*-values of these correlations are given in Table 3 (significant correlations are flagged with an asterisk).

**TABLE 3:** Correlations between NRT, PWR and reading.

Variable	Digit span	English NRT	NS Word reading	NS Text reading	English Word reading	English Text reading
NS NRT	0.34**	0.41**	0.44**	0.31**	0.27**	0.17
English NRT	0.54**	1	0.16	0.15	0.31**	0.33**
Digit span	1	0.54**	0.19*	0.22*	0.33**	0.33**

NRT, non-word repetition task; NS, Northern Sotho.

* *p* < 0.05;

** *p* < 0.01 (95% confidence interval)

Multiple hierarchical regression models with *NS word reading* and *NS text reading* as dependent variables and NS non-word repetition and digit span as predictors, showed that performance on the NS NRT reliably predicted the outcome of NS reading ability (non-word repetition was entered at Step 1 of the model and digit span was entered at Step 2). The ability of the NS NRT to predict English reading skills was not of primary interest here, and the correlations mentioned in Table 3 suggest that there is no, or a very weak relationship between the NS NRT and English reading. Thus, no regression with English reading as dependent variable is reported here. The constant values, betas, standard errors, standardised betas, and *R*^2^ values for each of these regression analyses are provided in Table 4.

**TABLE 4:** Hierarchical regression analyses with *NS Word reading* and, *NS text reading* as dependent variables and *NS NRT* and *digit span* as predictors.

Variable	NS word reading			NS text reading		
	
	B	SE	*Beta*	B	SE	*Beta*
**Step 1**						
(Constant)	6.94	9.74	-	6.99	9.07	-
NS NRT	0.86	0.16	0.44***	0.53	0.15	0.31**
**Step 2**						
(Constant)	4.72	17.04	-	-8.61	15.78	-
NS NRT	0.85	0.17	0.44***	0.46	0.16	0.27**
Digit span	0.18	1.14	0.01	1.28	1.06	0.11

NRT, non-word repetition task; NS, Northern Sotho; SE, standard error.

Note:  NS word reading: *R*^2^ = 0.195 for Step 1; Δ*R*^2^ for Step 2 = 0.000. NS text reading: *R*^2^ = 0.09 for Step 1; Δ*R*^2^ for Step 2 = 0.01. *, *p* < 0.05; **, *p* < 0.01; ***, *p* < 0.001 (95% confidence interval)

## Discussion

In South Africa, where many school-aged learners display below average language and literacy skills (Department of Basic Education, 2014; Howie, *et al*., 2008; Wilsenach, 2015), the dilemma of failing learners probably remains the most serious and most common problem that teachers, educational psychologists, and SLTs encounter. In this context, the accurate assessment of processing problems, particularly those related to language and literacy achievement is crucial. The current study set out to investigate the usefulness of using a NRT to detect phonological processing problems, indicative of reading failure, in the Northern Sotho-speaking population. Four research questions were posed, which will be discussed in this section. The first and second research questions (repeated below) will be discussed first, followed by a discussion of the third and fourth research questions:

- Does the NS NRT used in this study discriminate between Northern Sotho-speaking children with good and poor PWM skills?
- Does the LoLT affect the performance of Northern Sotho-speaking children on a NS NRT and on an English NRT?

The NS NRT developed and tested here clearly shows potential as a Northern Sotho language assessment instrument. The test scores obtained from a sample of 120 third graders were distributed normally, with scores ranging between 1 and 19 (0 being the lowest possible and 20 being the highest possible score). A visual inspection of the data confirmed a relatively flat and light-tailed distribution of scores, indicating that the test most certainly discriminated between learners with very weak and very strong NS phonological processing abilities. Both the Shapiro–Wilk test and the Q-Q plot suggested that the test scores were normally distributed, but a visual inspection of the data did suggest a slight build-up of higher scores (peaking around 65% correct responses). The ability of the test to discriminate between learners with ‘low average’, ‘high average’, and ‘moderately high’ phonological processing ability (if one is to assume a generic normal distribution curve) could most probably be improved on.

The longest non-words in the current version of the NS NRT consist of seven syllables, and all of the non-words follow the characteristic CVCV structure of Northern Sotho. Increasing the PWM load could be achieved by adding non-words of up to 10 syllables (which is the length of the longest English non-words in the CTOPP). It is, however, useful to consider other NRT design features in any revision of the current version of the NS NRT, as length is not the only complexity factor that can be introduced. Archibald and Gathercole (2006) compared performance on two English NRTs, namely the CNRep (Gathercole & Baddeley, 1996) and the NRT (Dollaghan & Campbell, 1998). Half of the non-words in the CNRep contain consonant clusters whereas the remainder have only single consonants. Many of the non-words in the CNRep include lexical components and morphemes, e.g. ‘pen’ in ‘pennel’/pεnᴉ/, and ‘ball’ in ‘ballop’/balǝp/. The CNRep non-words are presented with a typical English prosodic pattern. The non-words in the Dollaghan & Campbell task, on the other hand, contain a limited set of acoustically salient and early acquired phonemes (11 consonants and 9 vowels). The NRT non-words follow a CV structure, and none of the syllables correspond to English lexical items. Because of the absence of weak syllables, the prosody of these non-words is unlike the typical English prosodic structure. Summarising the features of the two tasks, one could say that the CNRep contains items that *sound more* like English words (and are higher in ‘wordlikeness’, because of the inclusion of lexical components and morphemes), but have greater articulatory complexity, whereas the NRT non-words *sound less* like English words, but are simpler in terms of articulation. (It should be noted that neither test contains lengthy non-words – the longest items in the CNRep are five syllables long, whereas the longest items on the NRT are four syllables long).

Memory for non-words is enhanced via existing knowledge of the lexical and phonotactic structure of a particular language (Roodenrys & Hinton, 2002; Vitevitch & Luce, 2005). Existing phonological representations of novel sound sequences can be activated via sublexical processing, which would support the processing of novel words (Martin & Gupta, 2004). According to the original PWM account of non-word repetition, proposed by Gathercole *et al*. (1994), children with impaired PWM are disadvantaged in repeating non-words as a result of the absence of lexical support. Thus, if a PWM deficit alone causes a non-word repetition deficit, children should be more disadvantaged on the NRT than on the CNRep, because of the greater presence of lexical and phonotactic knowledge-based support in the CNRep. However, Archibald and Gathercole (2006) found that children with SLI obtained lower scores on the CNRep, which they explained on the basis that children with limited vocabulary knowledge may be at a disadvantage in non-word repetition, not only because of impaired PWM, but also because they cannot support the temporary representations of non-words in their PWM with lexical and sublexical knowledge. Furthermore, the SLI children performed poorer in articulatory more complex non-words (i.e. non-word that contain consonant clusters). Possibly, these children have less robust phonological representations for relatively uncommon phoneme combinations. Alternatively, they may be less capable of producing stable rhythmic speech-motor movements, which may affect their ability to repeat consonant clusters. This explanation is supported by Klein, Watkins, Zatorre and Milner (2006), who found that distinct cortico cerebellar systems (associated with motor learning, and thus with the acquisition of the articulatory patterns of a language) are more active in the non-word repetition of English-French bilinguals when these individuals are asked to repeat complex words in their L2. Klein *et al*.’s findings suggest that increased articulatory demands imposed when producing novel sequences activate a cerebellar network, which requires more complex motor control for speech production.

It seems likely then that there are multiple origins to a deficit in non-word repetition, including PWM, lexical knowledge, and speech-motor processes, among others. An ideal NRT should include stimuli that address as many of these components as possible. It would therefore be advisable to not only add longer non-words to the current NS NRT, but to also carefully control the number and nature of articulatory more complex non-words, and to consider the use of items that can be categorised as ‘more wordlike’ and ‘less wordlike’. In the current version of the NS NRT, the non-words both *sound like* Northern Sotho words (i.e. are quite high in wordlikeness) and are *fairly simple* in terms of articulation. Increasing both length and articulation complexity should help to improve the power of the test to reliably distinguish between children who fall somewhere between a ‘low-average’ and ‘moderately-high’ profile in terms of Northern Sotho phonological processing ability.

The results of this study further suggest that Northern Sotho children, who receive their schooling in English, do not perform significantly differently on a NS NRT, compared to children who receive instruction in Northern Sotho. Group 2 scored higher on the NS NRT, but not significantly so, and thus the processing of phonological input in the L1 does not seem to be altered greatly in Northern Sotho children who receive their schooling in English. The first hypothesis of this study thus turns out to be incorrect. The two groups were also not different in terms of their ability to recall digits, suggesting that, at a very basic level, the groups’ cognitive functioning and PWM were alike. However, the LoLT did affect English phonological processing skills – learners who received their schooling in English from Grade 1 performed significantly better on the English NRT task. The poorer performance of the Northern Sotho LoLT group on the English NRT provides further support for the idea that performance on NRTs is not independent of language skill; the learners in Group 2 would have received far less linguistic input in English, and would probably have known significantly fewer English words. Thus, poor non-word repetition skills in bilingual populations is perhaps, like in clinical populations, the result of a poorly differentiated representational system arising from less effective lexical mediation.

The third and fourth research questions (repeated below) will be discussed next:

- Does the NS NRT used here correlate with other measures that tap into PWM, such as digit span and English NRT?
- Does the NS NRT used here predict word reading and fluent reading in Northern Sotho speaking children?

Pearson correlations on the collapsed data set established that performance on the NS NRT was significantly and positively associated with digit span (*R* = 0.34), with the English NRT (*R* = 0.41) and with both measures of Northern Sotho reading (*R* = 0.44 for word reading and *R* = 0.31 for text reading). The association between NS NRT and memory for digits was moderately weak. Memory for digits showed a moderately strong correlation with the English NRT. The association between the two NRTs was moderate, indicating that the tasks probably assessed the same underlying cognitive skill. Ideally, one would like to see stronger associations before reaching firm conclusions, but it is argued here that the NS NRT seems to be associated with both aspects conventionally associated with the PWM construct, namely the processing of information in the short term memory that have stored representations in long term memory and the processing of novel phonological representations which only simulate existing phonological structures.

Although this study aimed to test the ability of a NS NRT to identify phonological processing deficits in the Northern Sotho-speaking population at large (i.e. not taking into account the LoLT of a child), it is interesting to note that cross-linguistic PWM correlations were much stronger in the L1 instruction group than in the L2 instruction group. In the Northern Sotho instruction group, the NRTs showed a strong correlation with one another (*R* = 0.64), and with digit span (*R* = 0.51 for NS NRT and *R* = 0.59 for English NRT). In the English instruction group, the NRTs showed a weak correlation with one another (*R* = 0.28) and a weak correlation with digit span (*R* = 0.25 for NS NRT and *R* = 0.28 for English NRT). The NS NRT was moderately associated with Northern Sotho word reading, and weakly associated with Northern Sotho text reading and English word reading. This outcome echoes existing research, suggesting that non-word repetition is particularly associated with word reading (Botting & Conti-Ramsden, 2001; Baird, Slonims, Simonoff & Dworzynski, 2011). Interestingly, there were no significant correlations between English non-word repetition and Northern Sotho reading in the current study, which suggests a different cross-linguistic correlational pattern to the one reported in Veii and Everatt (2005), who found that L2 non-word repetition skills predicted more variance in L1 and L2 literacy than L1 non-word repetition.

Overall, the correlational pattern seems to suggest that phonological processing ability in bilingual children does not necessarily or automatically transfer across languages (i.e. high performance on a PWM measure in one’s L1 does not guarantee high performance on the same (or another) PWM measure in one’s L2. This notion is further supported by the absence of any meaningful associations between the English NRT and Northern Sotho reading, and between the NS NRT and English reading. The observed differences between the two groups with regards to the strength of cross-linguistic correlations seem to indicate that the LoLT affects the likelihood of transfer of certain aspects of linguistic knowledge. Northern Sotho learners who received their first years of schooling in their home language seem more likely to transfer phonological processing skills from their L1 to their L2, whereas Northern Sotho learners who received their schooling in English might not automatically transfer phonological processing skills acquired in English to Northern Sotho. However, a more systematic analysis of the data is required in order to disentangle the nature of transfer of linguistic knowledge in these groups of learners, and this was not the primary focus here.

In line with the initial finding of Wilsenach (2013), performance on the NS NRT reliably predicted word reading and fluent text reading in Northern Sotho in this study. Reading performance was clearly influenced by the LoLT, with Group 1 displaying stronger English reading skills, and Group 2 showing stronger Northern Sotho reading skills. NS NRT accounted for 19.5% of the variance in NS word reading, but only for 9% of the variance in fluent reading in the population at large. Memory for digits, which was entered as predictor of word reading and text reading in the second step of the regression model, made no independent contribution to performance in Northern Sotho reading, suggesting that the NS NRT tested here is a better indicator of reading performance than a digit span task. As a stand-alone assessment instrument, NRT is probably not powerful enough to detect problems in fluent reading in the population at large. Even so, 48 of the learners (40%) obtained 50% or less on the test, indicating that, unlike Bishop *et al*.’s (2014) claim, a substantial number of Northern Sotho children in the population at large seems to have compromised phonological processing abilities. The results presented here clearly show that non-word repetition distinguishes between learners with weak and strong PWM skills, and that the task is associated with reading performance on various levels. Thus, a NRT should form part of a test battery that measures Northern Sotho phonological processing. Other measures in a comprehensive test would include measures of phonological awareness, such as syllable and phoneme awareness as well as measures of phonological recoding, such as rapid naming.

## Conclusion

In the South African basic education context, where many learners fail to acquire literacy skills at an accepted standard, it is crucially important to be able to test phonological processing skills, in a child’s first language and in the LoLT. This article demonstrates that the developed NS NRT is a useful instrument, in that it reliably identifies children with poor PWM (and hence poor phonological processing) skills – such children are, by default, at risk of reading failure. The current version of the NS NRT can be improved on, specifically by adding longer and articulatory more complex non-words. Once this has been done, it would be worthwhile to standardise the test.

NRTs can be developed in all the African languages spoken in South Africa, and would, if designed properly, detect children with impaired phonological memory more reliably than other PWM such as digit and word span. The task is easy to administer and economical in terms of assessment time. Developing NRTs as stand-alone tests, or preferably as subtests in comprehensive phonological processing tests should thus receive more attention in the South African SLT, psycholinguistic, and educational psychology environment.

## Footnotes

1 The rationale for including Grade 3 learners was that the researcher wanted to test reading fluency in both Northern Sotho and English, which is often not possible before Grade 3 in this kind of research setting. Furthermore, the learners were assessed on a range of phonological skills (not reported on here) which were deemed too challenging for younger learners.

2 In order to determine the allocation of financial resources, South African public schools are categorised into five groups (quintiles). Quintile one schools are the ‘poorest’, while quintile five schools are the ‘least poor’. The poverty of the community surrounding the school and various infrastructural factors determine the ranking of a school.

3 The researcher opted to use only an English digit span task, as the participants in group 1 might not have had much exposure to the Northern Sotho equivalents of the digits 1–10. The participants in group 2, on the other hand, would have had ample exposure to these digits in English.

## Appendix A

### Northern Sotho non-word repetition task

1. Sêlumaka
2. Mofugatsadi
3. Balobadikwe
4. Basetswegokoela
5. Kuratshifodiri
6. Nthufobila
7. Katôngwaloshane
8. Batêraphôtwana
9. Hlôdikilêswagoba
10. Neratomkibangwane
11. Sepokari
12. Ntombuweka
13. Makepodiri
14. Nôrakulêswibisi
15. Bosithirangwe
16. Nasibhêkarabilê
17. Nesodiwako
18. Mogisiroletha
19. Hlatoyana
20. Narulongwakhubasi

## Appendix B

### Northern Sotho word reading test

1. nna
2. ema
3. tee
4. moo
5. eng
6. bona
7. yena
8. dira
9. kudu
10. fase
11. batho
12. mahlo
13. leina
14. phela
15. swara
16. ngwana
17. mathomo
18. meetse
19. bolela
20. morena
21. gopola
22. bošego
23. mantšu
24. kgopela
25. batswadi
26. hlodimela
27. monotlwana
28. phaphamala
29. tshesepere
30. gosenaselo
